# Experimental and numerical perspective on the fire performance of MXene/Chitosan/Phytic acid coated flexible polyurethane foam

**DOI:** 10.1038/s41598-021-84083-2

**Published:** 2021-02-25

**Authors:** Bo Lin, Anthony Chun Yin Yuen, Timothy Bo Yuan Chen, Bin Yu, Wei Yang, Jin Zhang, Yin Yao, Shuying Wu, Chun Hui Wang, Guan Heng Yeoh

**Affiliations:** 1 School of Mechanical and Manufacturing Engineering, University of New South Wales, Sydney, NSW 2052 Australia; 2 School of Energy, Materials and Chemical Engineering, Hefei University, Hefei, 23061 Anhui People’s Republic of China; 3 Centre for Future Materials, University of Southern Queensland, Toowoomba, QLD 4350 Australia; 4 School of Engineering, Macquarie University, Sydney, NSW 2109 Australia; 5 Australian Nuclear Science and Technology Organisation (ANSTO), Kirrawee DC, NSW 2232 Australia

**Keywords:** Two-dimensional materials, Synthesis and processing, Computational methods, Nanocomposites

## Abstract

Recent discoveries of two-dimensional transitional metal based materials have emerged as an excellent candidate for fabricating nanostructured flame-retardants. Herein, we report an eco-friendly flame-retardant for flexible polyurethane foam (PUF), which is synthesised by hybridising MXene (Ti$_3\hbox {C}_2$) with biomass materials including phytic acid (PA), casein, pectin, and chitosan (CH). Results show that coating PUFs with 3 layers of CH/PA/Ti$_3\hbox {C}_2$ via layer-by-layer approach reduces the peak heat release and total smoke release by 51.1% and 84.8%, respectively. These exceptional improvements exceed those achieved by a CH/Ti$_3\hbox {C}_2$ coating. To further understand the fundamental flame and smoke reduction phenomena, a pyrolysis model with surface regression was developed to simulate the flame propagation and char layer. A genetic algorithm was utilised to determine optimum parameters describing the thermal degradation rate. The superior flame-retardancy of CH/PA/Ti$_3\hbox {C}_2$ was originated from the shielding and charring effects of the hybrid MXene with biomass materials containing aromatic rings, phenolic and phosphorous compounds.

## Introduction

Polyurethane foam (PUF) has been utilised extensively for building and construction application (i.e. upholstery filler, walls and ceiling paddings, wall insulation boards)^1–3^, owing to its unique hyper-foam structure and versatile functionalities. It possess numerous advantageous properties such as superior compression strength, impact resistance, and softness^4,5^. Nevertheless, PUF also presents significant fire risks to human lives, properties, and environment due to their intrinsic high flammability and low ignition temperature^6–8^. In addition, the combustion process of PUF produces dangerous amounts of toxic and asphyxiant gases, such as carbon monoxide and cyanides^9,10^. Therefore, a wide range of flame-retardant (FR) additives and coatings have been developed to enhance the fire retardancy of PUFs, including inorganic and/or organic FRs^11–17^, as well as organic–inorganic hybrid FRs^18–21^. Although existing FRs can substantially reduce the flammability, the majority of these FRs, such as halogen-, and phosphorus-containing compounds are toxic and not biodegradable. Furthermore, the establishment of new regulations and standards restricting the use of these toxic FRs have also indicated the demand for a more eco-friendly and toxic-free FR^22^.

Another major issue with existing methods of bulk mixing FRs with the constituent compounds of PUFs is that desirable physical properties, such as softness, abrasion-resistance, and compression strength are inadvertently compromised^23–25^. To address this challenge, alternative methods for introducing FRs to the PUFs without negatively affecting the other important properties have been reported, including FR surface treatments, which modify only the outer surfaces of the PUF and avoid the shortcomings of bulk mixing^26,27^. Common surface coating methods include spinning, sputtering and chemical vapour deposition^28–31^. Recently, the layer-by-layer (LbL) assembly technique, which can deposit different functional layers of FRs on the PUFs without altering the bulk materials’ mechanical properties, has been applied to increase the fire safety of the PUF with less weight gain and additional functionalities^32–35^. The basic approach of the LbL assembly is to attach different functional coating materials onto the surface of the PUF, one layer after another by attraction forces, such as electrostatic, Van der Waals, and hydrogen-bonding^36,37^. The LbL deposition has been used to improve the thermal degradation resistance of cotton fabric with polyethylenimine and clays^38^, and PUFs using different organic and inorganic FR combinations^39–41^. Although reductions in the peak heat release rate (pHRR) were impressive, other properties such as smoke reduction, toxic gas suppression, and charring capability were less than satisfactory^42–45^. In addition, these coatings resulted in excessive weight gain compared to the pristine PUF. The present authors have recently combined a newly 2D nanosized material named MXene with chitosan (CH) to construct nanostructured FR coating for reducing the flammability of PUFs by using the LbL self-assembly method^46^. MXene refers to the 2D transition metal carbides or nitrides, which is commonly fabricated via etching from the precursor MAX phase materials, where “M” stands for the early transition metal, “A” represents the third or fourth group element and “X” is the carbon or nitrogen. MXene possesses superior electrical conductivity, which enables this material to have a very broad range of applications. Our previous findings showed that coating PUF with eight layers of CH/MXene (i.e. 8 monolayers of CH and 8 monolayers of MXene) significantly lowered the pHRR, total heat release (THR), total smoke release (TSR)^46^. Despite the outstanding results, the use of CH in the synthesis of CH/MXene coating, which has a relatively low FR efficiency, inspired the present research to hybridise MXene (Ti$_3\hbox {C}_2$) with other organic compounds, such as phytic acid (PA), pectin and casein, to achieve ultra-high performance while reducing the use of MXene.

**Figure 1:**
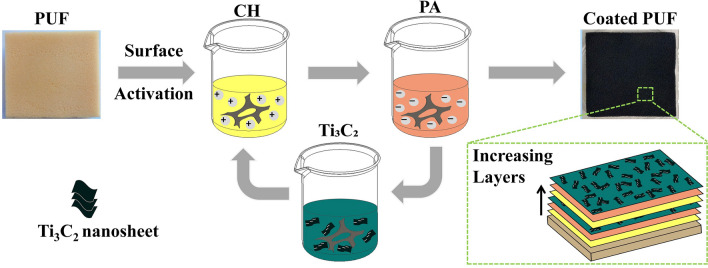
Schematic diagram of the fabrication process of CH/PA/Ti$_3\hbox {C}_2$ coated PUF via LbL approach.

The work reported herein is aimed at assessing the effectiveness of other biomass materials with high carbon content to improve the charring performance and subsequentially suppress the smoke and toxicity generations of PUFs. These biomass materials include casein, pectin, and PA. A systematic approach of the synthesis of CH/PA/Ti$_3\hbox {C}_2$ coating process for PUFs is given in Fig. 1. The flammability and asphyxiant gas releases of the FR-coated PUF were investigated through a series of benchmark fire tests and material characterisation techniques. To bridge the knowledge gap in the understanding of the underlying FR mechanisms, an analysis model was developed using computational fluid dynamics (CFD) techniques with an in-house surface regression pyrolysis module. This model fundamentally describes the flaming behaviour involving thermal degradation of the solid, charring, and combustion of the coated PUFs.

## Experimental results

### Characterisation of coated PUFs

**Figure 2:**
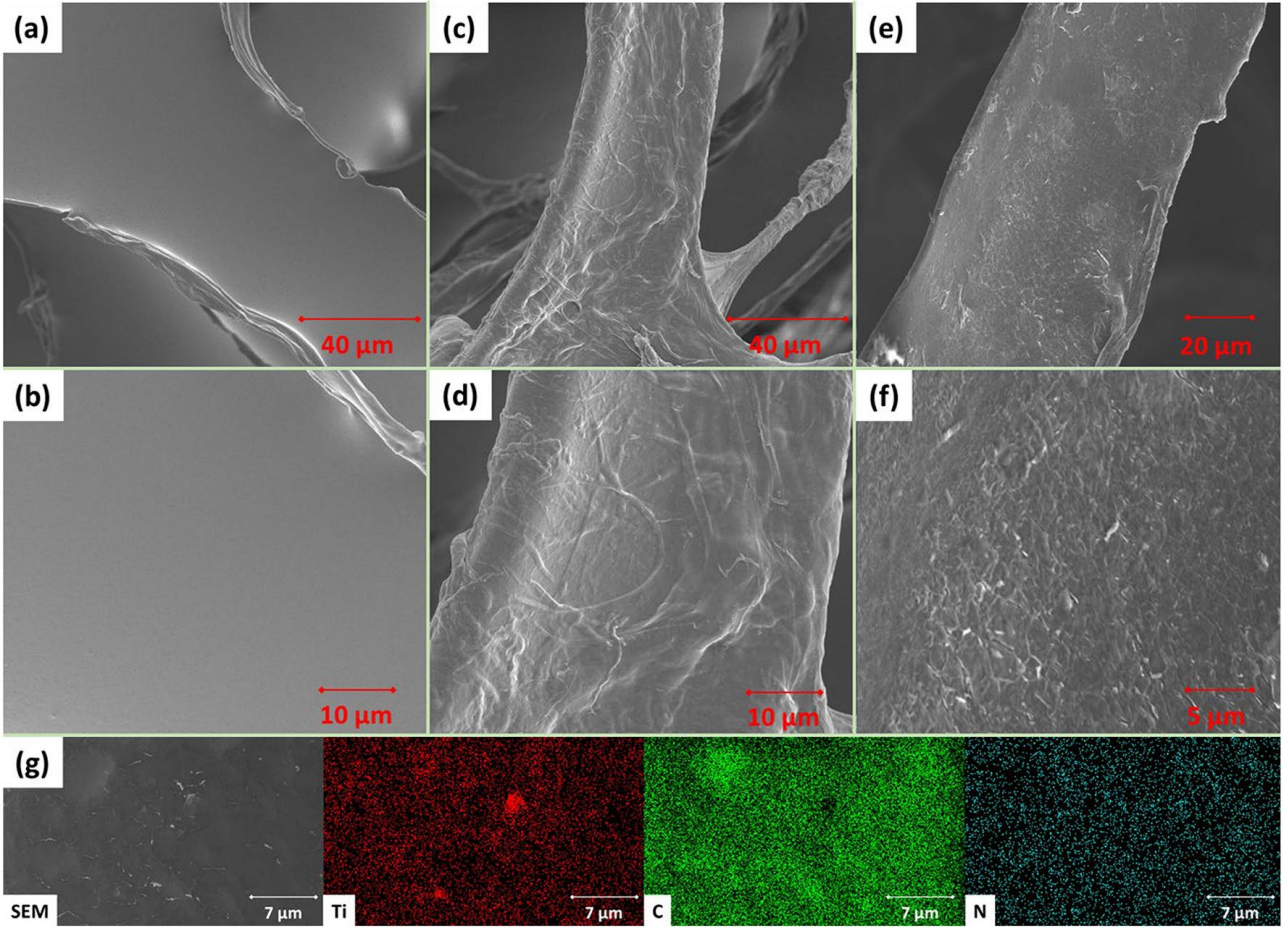
SEM images of (**a**,**b**) neat PUF, (**c**,**d**) [CH/PA]$_1$ coated PUF, and (**e**,**f**) [CH/PA/Ti$_3\hbox {C}_2$]$_1$ coated PUF, and (**g**) EDS mapping image of [CH/PA/Ti$_3\hbox {C}_2$]$_1$ coated PUF.

Morphology of the original PUF and coated PUFs using single layer of CH/PA and CH/PA/Ti$_3\hbox {C}_2$ was revealed via scanning electron microscopy (SEM) (Fig. 2a–f). The energy dispersive X-ray spectrometer (EDS) coupled to the SEM was employed to identify the elements on the surfaces of the CH/PA and CH/PA/Ti$_3\hbox {C}_2$ coated PUFs (Fig. 2g and Supplementary Fig. S1). It is clear from Fig. 2a,b that the original PUF exhibits a smooth surface without any discernible features. Figure 2c,d show the internal skeleton structure of the CH/PA coated PUF remained unchanged while the surfaces were fully covered with random patterns, indicating excellent attachment of the organic materials to the inner branch surfaces of the PUF. In addition, crosslinking patterns were visible between the branches in Fig. 2c, which is attributed to the chemical bonds between the CH long chains and PA. Moreover, compared with CH/PA coated foam, a smoother surface can be observed for the CH/PA/Ti$_3\hbox {C}_2$ coated PUF (Fig. 2e,f), which was due to the small dimensions and fine structure of the $\hbox {Ti}_3\hbox {C}_2$ nanosheets. However, when observed at a higher magnification, the surface appears to be rough with a wrinkle-like pattern of the MXene nanosheets (Fig. 2f). These corrugated surfaces illustrate the tight, uniform, and compact coating of the hybrid FR nanosheets that are deposited on the foam surfaces without any gaps or cracks. From the EDS image of the CH/PA/Ti$_3\hbox {C}_2$ coated PUF (Fig. 2g), it is clear that the Ti element possesses the same feature with the corresponding SEM image, which confirms that the Ti$_3\hbox {C}_2$ nanosheets have fully covered the surface of the PUF. Element mapping peaks of C, N, O and P are found in CH/PA coated PUF, and C, N, O and Ti peaks are displayed in CH/PA/Ti$_3\hbox {C}_2$ coated PUF (Supplementary Fig. S1). These element peaks observed in the Supplementary Fig. S1 further proved the existence of CH/PA and CH/PA/Ti$_3\hbox {C}_2$ coatings, which is consistent with SEM images (Fig. 2c–f). Based on the morphologies displayed in Fig. 2 and the difference between the CH/PA and CH/PA/Ti$_3\hbox {C}_2$ coated foam surfaces, it can be concluded that the CH, PA, and Ti$_3\hbox {C}_2$ were tightly, uniformly coated on the PUF surfaces, including the surfaces of the inner skeleton structure, with no gaps or defects.

### Thermal stabilities of PUFs

**Figure 3:**
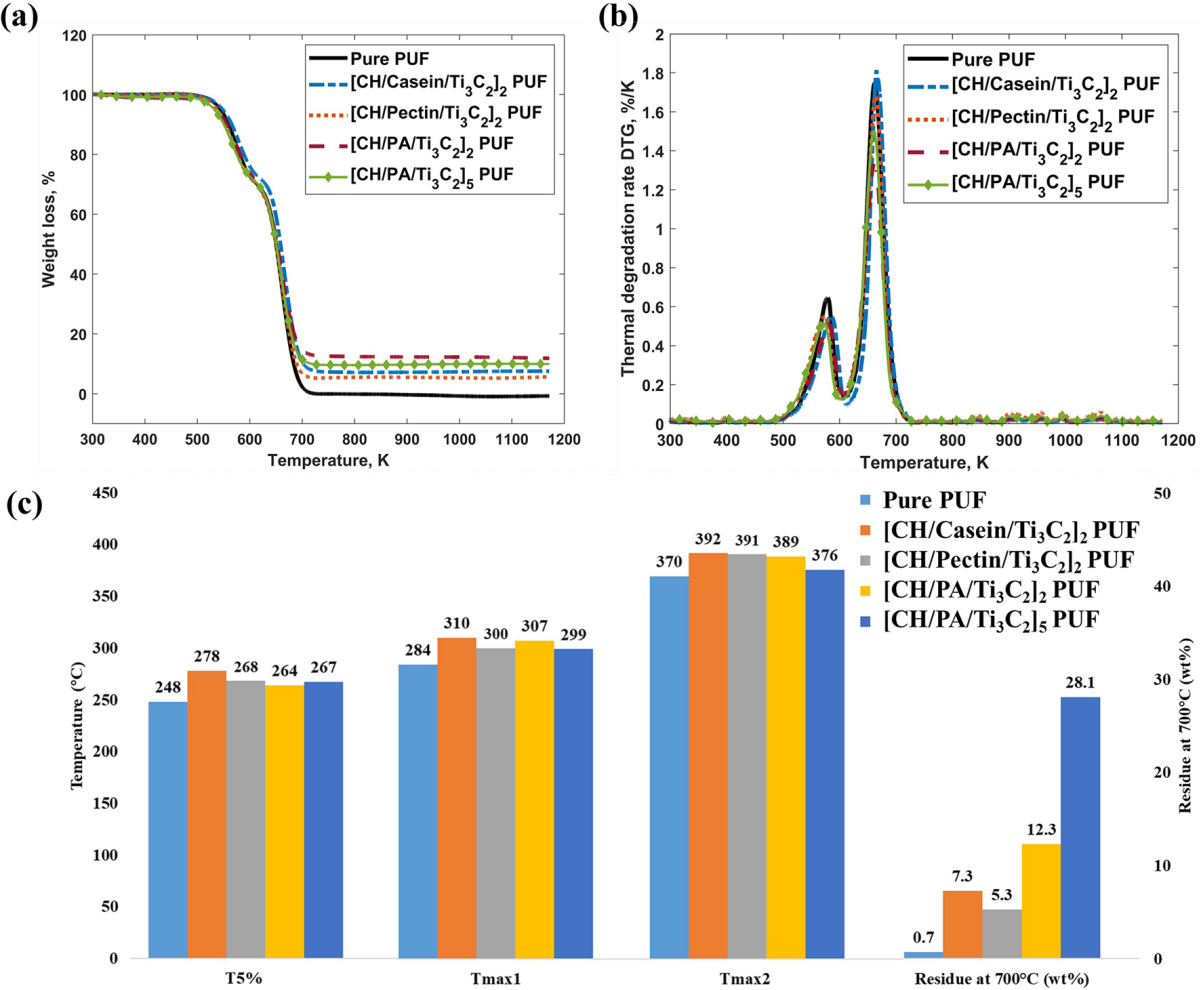
(**a**) TG, (**b**) DTG and (**c**) summarised TGA results of neat PUF and PUF coated with CH/casein/Ti$_3\hbox {C}_2$, CH/pectin/Ti$_3\hbox {C}_2$, and CH/PA/Ti$_3\hbox {C}_2$.

Thermal degradation and differential thermal degradation curves of the neat PUF and the PUFs coated with different hybrid organic-Ti$_3\hbox {C}_2$ nanosheets (2 and 5 layers) are shown in Fig. 3a,b. The temperatures of different degradation stages for all samples are summarised in Fig. 3c. Actual values of the temperatures, weight gains and the residues at 700 $^\circ$C are summarised in Supplementary Table S1. In this work, [CH/PA/Ti$_3\hbox {C}_2$]$_n$ refers to the resultant coating with “n” denoting the number of layers. Further explanation of this naming convention is illustrated in the “Methods” section. The start of thermal degradation is commonly characterised by the temperature T$_5$%, which refers to the temperature at which the samples lose 5% of their initial weight. According to Fig. 3b, all the PUF and its composites exhibit two decomposition peaks, corresponding to two degradation stages labelled as T$_{max1}$ and T$_{max2}$, respectively. For the original foam, the thermal degradation began at 248 $^\circ$C (T$_5$%), with the first degradation occurring at 284 $^\circ$C (T$_{max1}$) and a peak decomposition process at 370 $^\circ$C (T$_{max2}$). The residue of the original foam at 700 $^\circ$C was found to be 0.7 wt%. The first stage of the original PUF thermal degradation is attributed to the thermolysis of the urethane, resulting in the substituted urea groups. This corresponds to the small peak with a mass loss rate of around 25 wt%. The second stage of thermal degradation was due to the pyrolysis of the remained components, which was accompanied by a weight loss of approximately 74%. During the second stage, all the remaining components of the foam started to decompose, resulting in the most substantial and apparent weight loss. Finally, at a temperature of 700 $^\circ$C, all the remaining components were carbonised, with a minute char yield of 0.7 wt%. Compared to the pristine foam, all the coated foams showed delays in the initial temperature of degradation (Fig. 3). In addition, for all these coated foams, the degradation rates decreased, and the two decomposition peaks occurred at higher temperatures, confirming the improvement of thermal resistance^47^. This is attributed to the high thermal conductivity of Ti$_3\hbox {C}_2$ nanosheets, which improve the heat transfer evenly along the surface and slow the heat penetration to the under layers^48^. This mechanism also postpones the degradation of the PUF composites and leads to a barrier effect that fullly protects the PUF from heat and pyrolysis. All the flame retarded foams yielded higher residues at 700 $^\circ$C with values of 7.5, 5.3, 12.3 wt% for the foams coated with CH/casein/Ti$_3\hbox {C}_2$, CH/pectin/Ti$_3\hbox {C}_2$, and CH/PA/Ti$_3\hbox {C}_2$, respectively, which are higher than the char yield of the original PUF. However, when considering the weight gain to the pure PUF, only CH/PA/Ti$_3\hbox {C}_2$ coated PUFs demonstrated improvement of the residue values (Supplementary Table S1). Besides, from the TGA traces, it was found that CH/PA/Ti$_3\hbox {C}_2$ coating imparted the PUF with the best thermal stability. Interestingly, PUFs coated with two or five layers of CH/PA/Ti$_3\hbox {C}_2$ (i.e. [CH/PA/Ti$_3\hbox {C}_2$]$_2$ and [CH/PA/Ti$_3\hbox {C}_2$]$_5$), showed similar thermal stability, which suggests that increasing the CH/PA/Ti$_3\hbox {C}_2$ coating beyond two layers yields little further improvement of the thermal resistance, although the residuals seem to increase linearly with the number of layers.

### Microscale cone calorimetry and UL-94 Horizontal burning test

**Figure 4:**
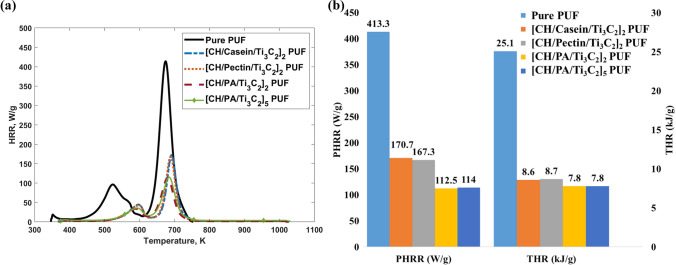
(**a**) HRR curves and (**b**) summarised MCC results for pure and all coated PUFs.

Microscale combustion calorimeter (MCC) is a commonly used benchmark test to study the combustion characteristics of PUFs^49,50^. It is a small scale method to investigate the combustibility and FR properties of polymeric materials^51^. The pHRR and THR results of all the neat and coated PUFs are shown in Fig. 4. The actual values of the pHRR and THR were summarised in Supplementary Table S2. Two peaks are found for all the curves (Fig. 4a), which is consistent with the two degradation stages of the foams as observed in the TGA results. For the pure PUF, these two peaks are wide and significantly high, corresponding to the large oxygen consumption with a pHRR value of 413.3 W/g and a THR value of 25.1 kJ/g. For all the coated foams, it is found that the first peak heat release temperatures shifted higher by around 50$^\circ$, demonstrating that higher temperatures are needed to trigger the combustion (Fig. 4a). Moreover, it was found that, compared with the neat PUF, all the PUF composites display much lower heat release rate (HRR) values at both two peaks (Fig. 4b). Also, it should be noted that for the CH/PA/Ti$_3\hbox {C}_2$ coated foam, the reductions in both pHRR and THR were the highest among all coated foams with values of 73% (112.5 W/g) and 69% (7.8 kJ/g), respectively. This is due to the synergistic charring effect of the nanostructured FRs. The outer coating layers containing abundant organic materials were first to degrade before the PUF, which results in the first heat release peak. Simultaneously, the degradation of the Ti$_3\hbox {C}_2$ layers inside the LbL assembly leads to the formation of tight and dense TiO$_2$ “shield” layers that fully cover the organic materials from heat and oxygen. This behaviour delayed the combustion and lowered the HRR of the first peak. Subsequently, as the temperature increased, more char was formed which not only acted as a thermal barrier against the heat but also isolated the combustible volatiles from the outside oxygen environment. These contributed to the delayed combustion and the lower second peak. In addition, further investigation and discussion regarding the synergistic effects of the coatings were performed and illustrated in the Supplementary Documents (Supplementary Table S3). Among all the FR coatings, the CH/PA/Ti$_3\hbox {C}_2$ yielded the best flame retardancy performance with the lowest pHRR and THR values. Comparing the results of [CH/PA/Ti$_3\hbox {C}_2$]$_5$ with [CH/PA/Ti$_3\hbox {C}_2$]$_2$, an increase in the number of layers from two to five yielded no further improvement.

**Table 1:** UL-94 horizontal burning test results of all the PUFs.

Sample	Pure foam	[CH/Casein/Ti$_3\hbox {C}_2$]$_2$ PUF	[CH/Pectin/Ti$_3\hbox {C}_2$]$_2$ PUF	[CH/PA/Ti$_3\hbox {C}_2$]$_2$ PUF
Dripping	Yes	No	No	No
Burning rate (mm/min)	237 ± 15	360 ± 15	331 ± 22	173 ± 11

**Figure 5:**
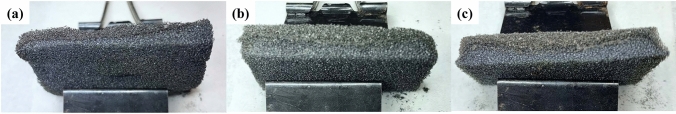
Cross-section digital images of UL-94 horizontal burning testing residues of (**a**) [CH/Casein/Ti$_3\hbox {C}_2]_2$, (**b**) [CH/Pectin/Ti$_3\hbox {C}_2]_2$, and (**c**) [CH/PA/Ti$_3\hbox {C}_2]_2$ coated PUFs.

To further investigate the ignitability and flammability of the PUF samples, the UL-94 horizontal burning tests were performed to the pristine, [CH/Casein/Ti$_3\hbox {C}_2]_2$, [CH/Pectin/Ti$_3\hbox {C}_2]_2$ and [CH/PA/Ti$_3\hbox {C}_2]_2$ coated PUFs according to ASTM D 4986, ISO/DIS 9772.3 standard. The results are summarised in the Table 1 and the cross-section digital images of the post-test PUF composite residues are shown in Fig. 5. During the test, neat PUFs were ignited quickly with large flame and smoke. The burning rate of pure PUFs is around 237 mm/min. No residues were left after the combustion of the pristine PUFs, and the drippings caused the cotton balls at the bottom to burn. For the [CH/Casein/Ti$_3\hbox {C}_2$]$_2$ and [CH/Pectin/Ti$_3\hbox {C}_2$]$_2$ coated PUFs, the flame was smaller, and the smoke was significantly suppressed. The burning rates increased to 360 and 331 mm/min, respectively, which is attribute to the high carbon contained organic coatings (CH, casein and pectin). No drippings were found after the tests and the samples were preserved well. During the horizontal tests of the [CH/PA/Ti$_3\hbox {C}_2$]$_2$ coated PUFs, the flame and smoke were further suppressed, and the burning rate was reduced to 173 mm/min. This reduction in the burning rate results from the high phosphorus groups contained in the PA that promotes the carbonisation and dehydration processes^52^. Together with Ti$_3\hbox {C}_2$, the char formation process was further improved, resulting in a decrease in the burning rate. In Fig. 5, a clear boundary between the char and the original coated foam can be found for all the coated PUFs, where the upper area are the char formed from the coatings and the PUFs beneath the coatings, and the lower area are the unburned coated PUF protected by the char. Therefore, the [CH/PA/Ti$_3\hbox {C}_2]_3$ PUF was further characterised in the cone calorimeter testing for a more comprehensive study of the flame retardancy.

**Figure 6:**
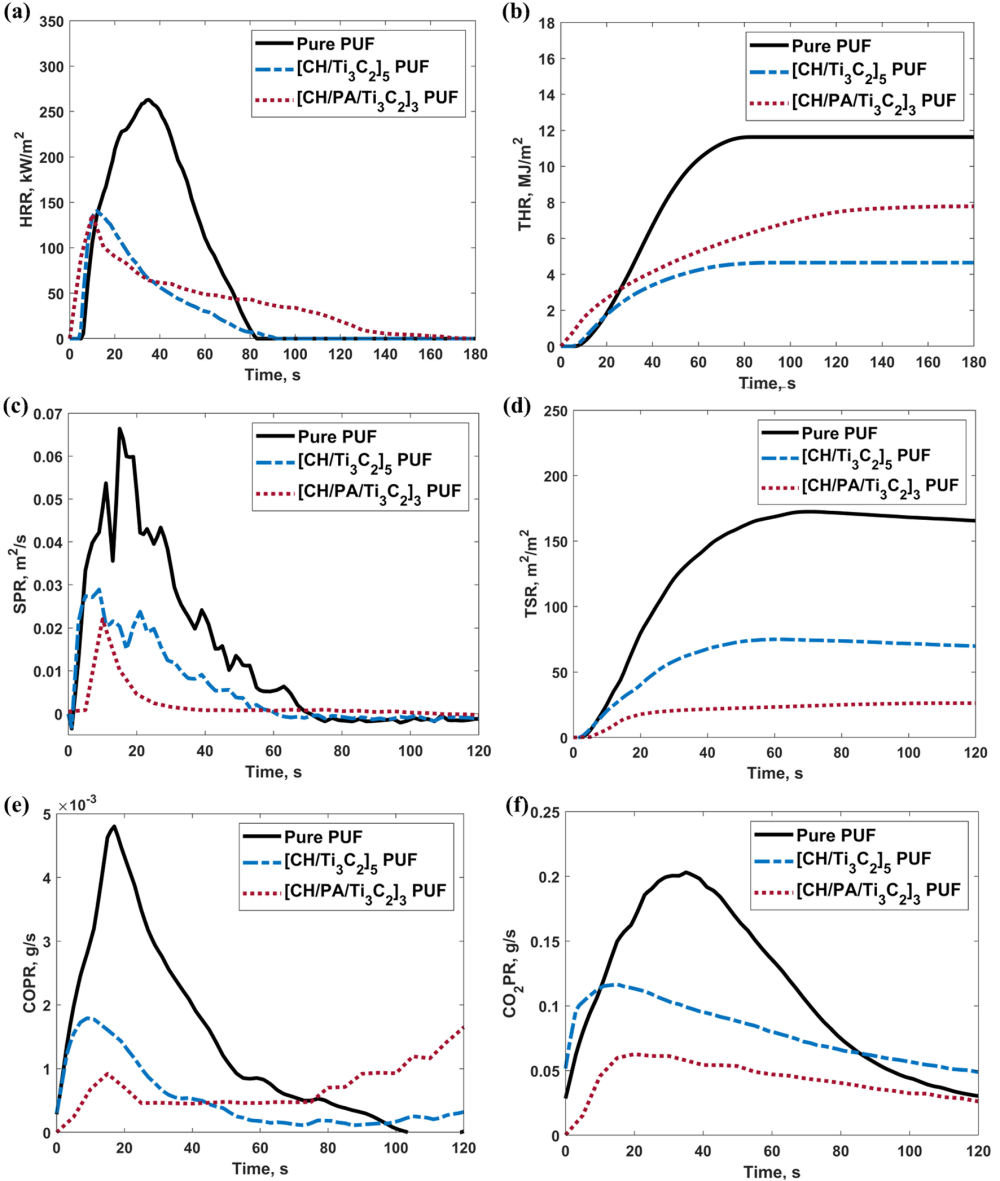
Cone calorimetry results of (**a**) HRR, (**b**) THR, (**c**) SPR, (**d**) TSR, (**e**) COPR and (**f**) CO$_2$PR of the pure and all coated PUFs.

### Cone calorimeter test

Cone calorimeter is employed to characterise the combustion performance of polymer materials by measuring the HRR based on the principle of oxygen consumption and other characteristics of the materials^53,54^. Since cone calorimeter provides the test environment close to the real combustion environment, the results can be used to evaluate the burning behaviour of materials in a fire^55,56^. This benchmark testing was conducted to demonstrate the fire safety performance of the CH/PA/Ti$_3\hbox {C}_2$ coated PUF. Key performance parameters include time to ignition (TTI), pHRR, THR, smoke production rate (SPR), TSR, carbon monoxide production rate (COPR), and carbon dioxide production rate (CO$_2$PR). The results compared with our previous work on CH/MXene are summarised in Supplementary Table S4 and displayed in Fig. 6. According to Supplementary Table S4, CH/PA/Ti$_3\hbox {C}_2$ increased the TTI by 1 s compared to the neat PUF (5 s). According to the HRR curves shown in Fig. 6a, the [CH/Ti$_3\hbox {C}_2$]$_5$ coating reduced pHRR from 276 kW/m$^2$ to 140 kW/m$^2$. By contrast, the [CH/PA/Ti$_3\hbox {C}_2$]$_3$ coating reduced pHRR to 136 kW/m$^2$, which represents a pHRR reduction of 51% compared with the pristine PUF.

The THR curve of the [CH/PA/Ti$_3\hbox {C}_2$]$_3$ PUF indicates a reduction of 35% compared with the neat PUF, which was slightly better than [CH/Ti$_3\hbox {C}_2$]$_5$ PUF due to the higher concentration of organic materials (Fig. 6b). These results show that CH/PA/Ti$_3\hbox {C}_2$ is more effective in improving the flame retardancy properties of PUF comparing to our previous work on CH/Ti$_3\hbox {C}_2$.

Apart from the heat release, smoke is also an essential factor, since toxic and asphyxiant gases cause huge, permanent, and even fatal damage to human bodies. Carbon monoxide (CO) and carbon dioxide (CO$_2$) are representative gases for benchmark testing. Because these gases, above certain amounts, can dilute the concentration of oxygen and cause asphyxia, which results in fatalities during escape and rescue. According to the SPR curves in Fig. 6c, the [CH/Ti$_3\hbox {C}_2$]$_5$ coating significantly reduced the SPR during the whole combustion period. By contrast, the [CH/PA/Ti$_3\hbox {C}_2$]$_3$ PUF was able to achieve a peak smoke production rate (PSPR) reduction of 68%, greater than the 54.4% achieved by [CH/Ti$_3\hbox {C}_2$]$_5$ PUF. In the TSR curves shown in Fig. 6d, the [CH/PA/Ti$_3\hbox {C}_2$]$_3$ coating demonstrated the highest reduction of TSR: 85% compared to the 56.6% achieved by [CH/Ti$_3\hbox {C}_2$]$_5$. This superior performance can increase the chances of survival during fire scenarios.

Results of the CO and CO$_2$ productions by the different PUFs, as shown in Fig. 6e,f, revealed that the [CH/PA/Ti$_3\hbox {C}_2$]$_3$ coating significantly delayed the release of CO and CO_2_ below [CH/Ti$_3\hbox {C}_2$]$_5$, albeit the CO level increased after 40 s. This is due to the high concentration of organic materials that leads to denser char formation, which creates a shielding effect to prevent the PUF from reacting with oxygen. Although the [CH/PA/Ti$_3\hbox {C}_2$]$_3$ PUF showed an increasing trend during the final stage of the burning, the reduction of the CO was still significant with a value of 60% compared with the pure PUF. According to the CO$_2$ release curves in Fig. 6f, the [CH/PA/Ti$_3\hbox {C}_2$]$_3$ coating showed the best performance in lowering CO$_2$, with a reduction of 68% in the CO$_2$P compared with the neat PUF.

In addition, a comprehensive investigation into the high fire safety performance in terms of the content of MXene was presented in the Supplementary Documents S4. FR properties of different MXene LbL coatings regarding the number of MXene monolayers was benchmarked. In terms of the number of MXene monolayers applied within the polymer matrix, it was identified that our present work significantly outperformed previous work in terms of pHRR, PSPR, TSR, PCOPR and PCO$_2$PR. This further signified the excellent performances of the MXene biomass nanocomposite. It also confirmed that while MXene was an effective FR, the optimal amount of monolayer is around 3, while the remaining could be replaced by alternative organic variants to improve the eco-friendliness.

Therefore, it can be concluded that the CH/PA/Ti$_3\hbox {C}_2$ is a significantly better and eco-friendlier FR coating than CH/Ti$_3\hbox {C}_2$ with excellent reductions in heat, smoke, and toxic gases, which will provide significantly better protection against fire, aid the escape and rescue, and improve the chances of survival.

**Figure 7:**
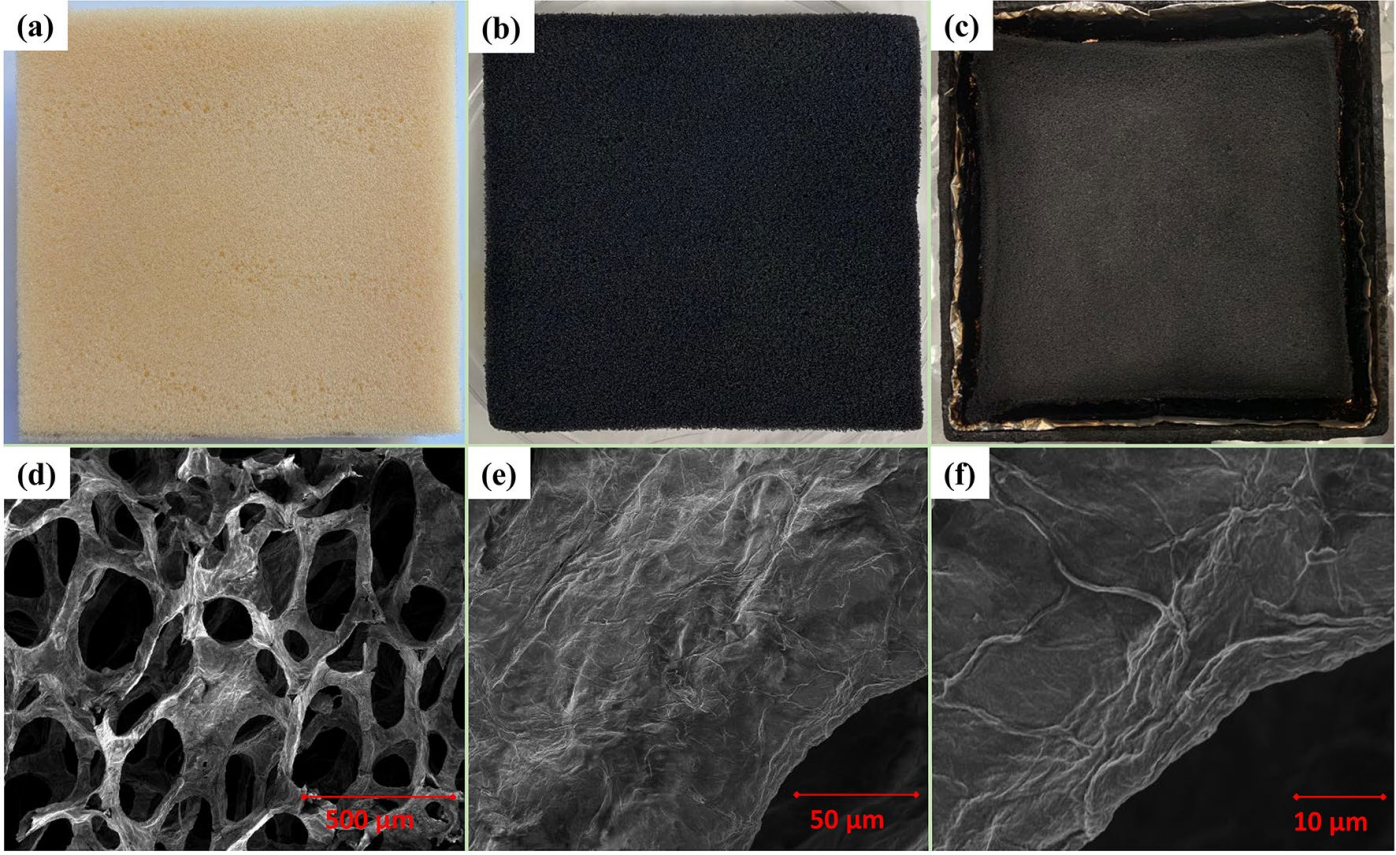
Digital images of (**a**) pure PUF, (**b**) [CH/PA/Ti$_3\hbox {C}_2$]$_3$ PUF, and (**c**) [CH/PA/Ti$_3\hbox {C}_2$]$_3$ PUF after Cone test; and SEM images of (**d**,**e**,**f**) [CH/PA/Ti$_3\hbox {C}_2$]$_3$ PUF after Cone test.

### Char forming performance

A comparison of digital photos of the pristine PUF and coated PUF, before and after burn is presented in Fig. 7a–c. It is clear that with [CH/PA/Ti$_3\hbox {C}_2$]$_3$ coating, the foams retained their original shape after burning due to the formation of chars. The SEM images in Fig. 7d–f show that the internal skeleton structure of the PUF was well preserved with no visible damage to the branches, although slight shrinkage of the branches was observed. In the higher resolution images, the remained organic and inorganic patterns remain clearly visible on the surface of the foam, suggesting a fine and compact char formation on the surface which fully covers the areas of the branches. The fine char layer formation not only prevents the combustible violates from migrating to the foam surface but also suppresses the generation of gaseous and smoke products. Furthermore, the char layer insulates the foam from the external heating environment, and thus results in the significant flame retardancy.

## Numerical results

CFD simulations were carried out based on the cone calorimeter experiment to further investigate the flaming behaviour and char formation of the CH/PA/Ti$_3\hbox {C}_2$ coated PUF. Numerical modelling provides additional theoretical insight into the transient flaming behaviours, chemical species and smoke concentrations^57^. The fire model was developed using ANSYS Fluent version 19.2, extending a three-dimensional porous media pyrolysis model from previous work^58^. The large eddy simulation (LES) based fire model takes into consideration of (i) solid pyrolysis, (ii) gas-phase combustion, (iii) radiation heat exchange between fire source, walls, and gaseous products, (iv) soot formation, and (v) sub-grid scale (SGS) turbulence models. The solid pyrolysis model was developed with user-defined functions (UDF) which describe the solid thermal degradation process and porous properties of the sample. The section below provides a detailed description of the pyrolysis model; details of the other modelling components such as combustion, radiation and soot formation can be found in the Supplementary Document.

### Porous media char formation pyrolysis model

The in-house pyrolysis model considers the rate of thermal degradation in the form of an Arrhenius expression. Each material component may undergo several competing reactions, and each of these reactions produces solid component (char) and gaseous species according to specified yield coefficients. The thermal degradation rate *R* of a material is the sum of all the reaction rates for gas volatiles $r_{g}$ and the char formation $r_{c}$ given by:

1 $$\begin{aligned} R=\, & r_g + r_c, \end{aligned}$$

2 $$\begin{aligned} r_{g}= & \sum _{i=1}^{l} (1-v_{residual})c_{i}A_{i}exp\left( -\frac{E_{i}}{RT_{s}}\right) (Y_{i})^{n_{i}}, \end{aligned}$$

3 $$\begin{aligned} r_{c}= & {} \sum _{i=1}^{l} (v_{residual})c_{i}A_{i}exp\left( -\frac{E_{i}}{RT_{s}}\right) (Y_{i})^{n_{i}}, \end{aligned}$$

where ${A_i}$ denotes the pre-exponential factor, $E_i$ the activation energy, $n_i$ the exponent factor, and ${c_i}$ the composition of each reaction. Parameter $v_{residual}$ is the residual mass fraction and $Y_i$ is the solid mass fraction. These parameters are commonly referred to as the pyrolysis reaction rate kinetics and are extracted from TGA measurements. To incorporate these pyrolysis reaction rates into the mathematical model, two new terms, $f_s$ (solid fraction) and $f_c$ (char fraction), have been introduced as transporting properties. The depreciation of the solid is governed by the rate of pyrolysis/solid decomposition *R* as specified in Eqs. (1), (2) and (3). According to these solid fractions, the material properties such as porosity and thermal conductivity changes according to the composition of solid (solid + char) to gas. The amount of gas volatile fuel releases, which is an input for the combustion model is given by:

4 $$\begin{aligned} \dot{m}_{fuel} = - \int _{0}^{H}\rho VR(y) \approx \int _{j=0}^{H}\rho Vr_{g}. \end{aligned}$$

In Eq. (4), the mass generation rate of the gas-volatile $\dot{m}_{fuel}$ is given as an integral of all reaction rates *R(y)* towards the depth of the solid *H* from the surface level y = 0. This model assumes: (i) the release of fuel is instantaneous, (ii) there are no moisture effects, (iii) all the pyrolysis gas releases occur at the surface of the fuel bed at y = 0 and the fuel is injecting normally against the surface. The integral can be approximated by the summation of all the reaction rates multiplied by the density $\rho$ and volume *V* of all the elements within the depth of the solid region.

### Pyrolysis reaction rate characterisation

The pyrolysis reaction rates (i.e. $A_i$, $E_i$, $n_i$ and $c_i$) for the numerical model were extracted from the derivative thermogravimetric (DTG) data via an iterative MATLAB code^59^. The procedure comprises of first calculating an initial estimation of the reaction rates using the Kissinger–Akahira–Sunose (KAS) method^60^. It is a well-established method for estimating the pyrolysis reaction rates based on a relationship between the heating rate (K/min) applied in the TGA and corresponding peak reaction rate temperature. The initial approximation obtained from the KAS method was then optimised by applying a genetic algorithm (GA) based optimisation method to achieve a better fit with experimental data. The GA process was repeated until convergence (i.e. no subsequent improvement in the fitness functions or error of less than 5%). The pyrolysis reaction rate parameters for the PUF samples are shown in Table 2. The two reaction rates (i.e. *R1* and *R2*) correspond to the two peaks in the DTG curve. Overall, the two peaks in the experimental curves were fully replicated by the numerical results generated by using the extracted reaction rate kinetics in the Arrhenius expression. More details on the pyrolysis characterisation method and comparison between the numerical and experimental DTG curves can be found in the Supplementary Document.

**Table 2:** Pyrolysis reaction rate kinetics for pure PUF and coated PUF.

Sample		E$_i$ (J mol$^{-1}$)	A$_i$ (s$^{-1}$)	c$_i$	n$_i$
[CH/PA/Ti$_3\hbox {C}_2$]$_3$ PUF	R1	2.369E+05	7.903E+20	0.25	2.2
	R2	1.906E+05	2.267E+13	0.75	1.2
Pure PUF	R1	1.204E+05	4.880E+09	0.36	1.0
	R2	2.144E+05	5.650E+15	0.64	2.0

### Cone calorimeter simulation results

**Figure 8:**
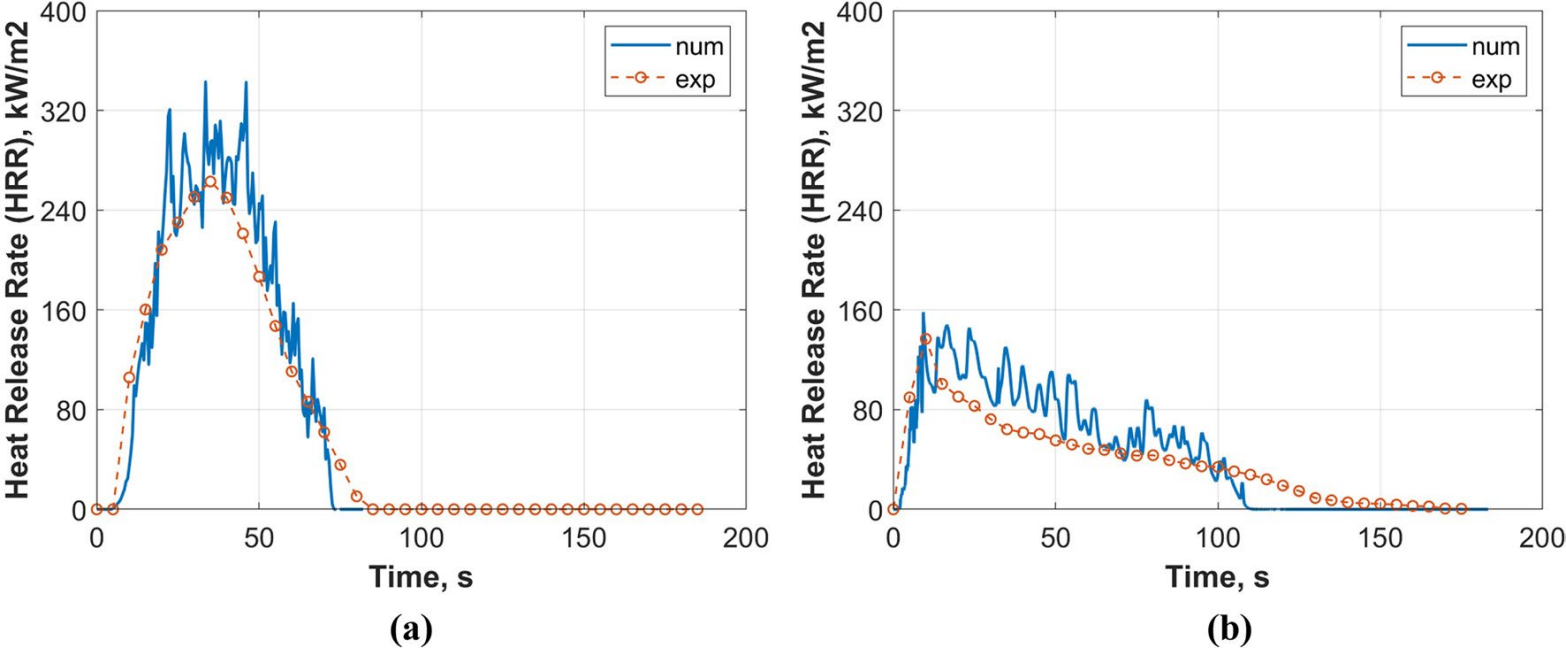
Experimental and numerical HRR from the cone calorimeter for (**a**) pure PUF and (**b**) [CH/PA/Ti$_3\hbox {C}_2$]$_3$ PUF under a heat flux of 35 kW/m$^2$.

Numerical simulations have been performed on two selected cases: (a) pure PUF and (b) [CH/PA/Ti$_3\hbox {C}_2$]$_3$ PUF, to compare the burning behaviour between the most effective coating and benchmark non-treated foam. The numerical simulation was validated against results from the cone calorimeter experiments. As shown in Fig. 8, the overall HRR profiles were successfully captured by the numerical simulation, with a strong single peak as observed experimentally. In terms of TTI, both the predictions for pure PUF and [CH/PA/Ti$_3\hbox {C}_2$]$_3$ PUF are in good agreement with the experimental results as the initial increase in HRR was correctly captured by the fire model. Nonetheless, the subsequent HRR profile after the initial jump was overpredicted. From experiments, the pHRR for pure PUF is 276 kW/m$^2$ occurring at approximately 34.85 s while the numerical prediction is 351.89 kW/m$^2$ (+ 27.50%) at 33.51 s (Fig. 8a). By contrast, the predicted pHRR for the [CH/PA/Ti$_3\hbox {C}_2$]$_3$ PUF is 144.39 kW/m$^2$ occurring at approximately 9.83 s compared to the 136 kW/m$^2$ at 9.30 s from the experiment, with a discrepancy of 6.17% (Fig. 8b). Both the burn duration and time to extinguish results from the simulations are also in line with the experimental data. The HRR profile from the experiments approaches zero at approximately 81.16 s and 174.89 s for pure PUF and [CH/PA/Ti$_3\hbox {C}_2$]$_3$ PUF, respectively, compared to 73.25 s (− 9.74%) and 109.7 s (− 37.2%) predicted by the model. The minor discrepancy in the predicted time for the [CH/PA/Ti$_3\hbox {C}_2$]$_3$ PUF might be attributed to the following factors: (i) there are discrepancies in the thermal behaviour between TGA results and cone calorimetry, especially for materials with LbL coating. (ii) the numerical model does not take into account any smouldering effects which might extend the HRR profile in the experiment. Nonetheless, the overall results demonstrate that the numerical model incorporating the pyrolysis reaction rates extracted from the GA approach was able to accurately capture the fire development of the PUF with and without FR coating. This new computational model offers advanced simulation capability for further design and optimise FR coatings.

**Figure 9:**
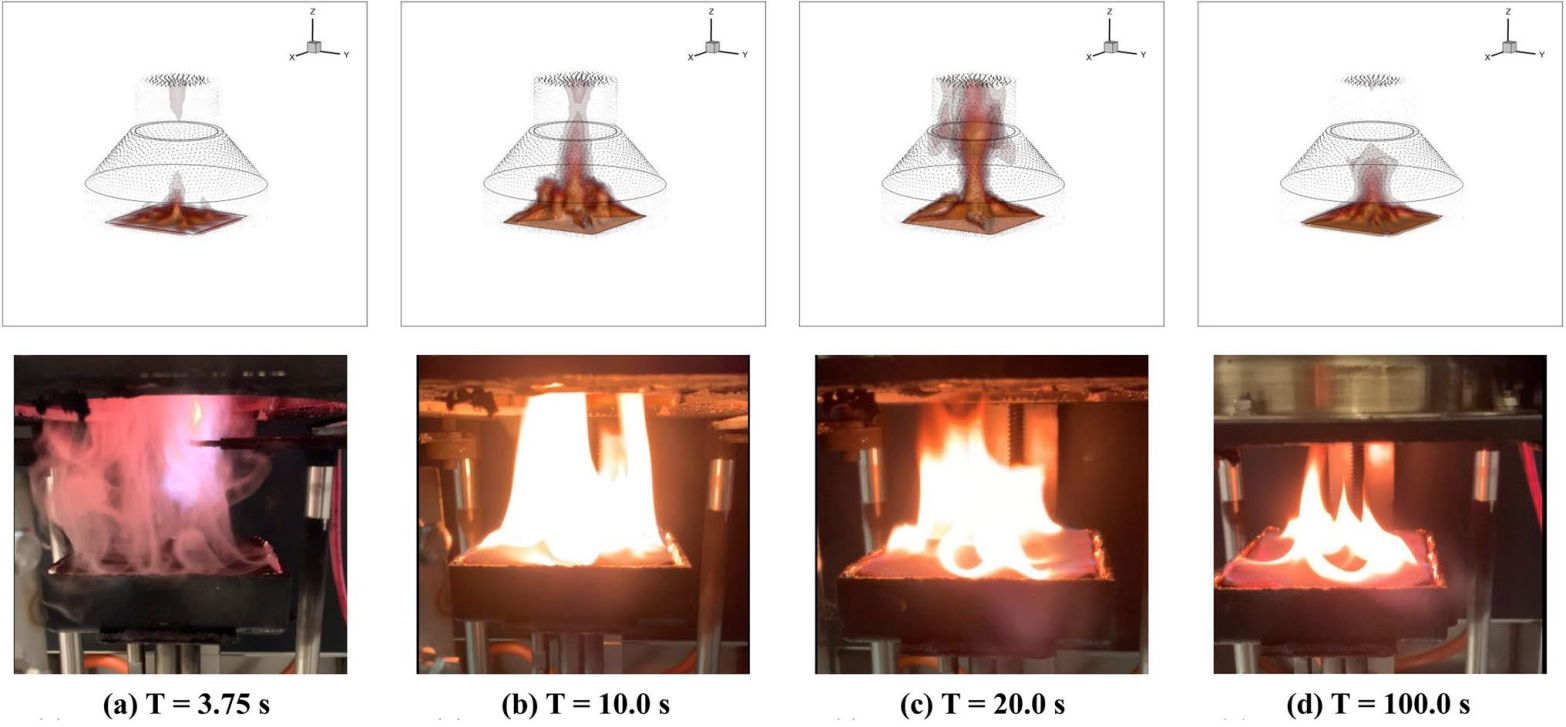
Three dimensional flame visualisation for 35 kW/m$^2$ cone calorimeter simulation and experimental image for [CH/PA/Ti$_3\hbox {C}_2$]$_3$ PUF at time instance: (**a**) T = 3.75 s, (**b**) T = 10.0 s, (**c**) T = 20.0 s, and (**d**) T = 100 s.

By applying the three-dimensional LES modelling approach, the full duration of the cone calorimeter test can also be visualised by using iso-surface plots. The 3D flame visualisation of the cone calorimeter experiment of the PUF coated with [CH/PA/Ti$_3\hbox {C}_2$]$_3$ at several time instances are illustrated in Fig. 9a–d. The images were generated using isometric contour plots with a gas temperature at 500 $^\circ$C (in red) which corresponds approximately to the temperature cut off for visible flame. As can be seen in Fig. 9a, the first instance of visible flame is approximately at T = 3.75 s. During this first stage, the pyrolysis rate gradually increases as the solid fuel is heated up and the flame is fully developed by T = 10 s (Fig. 9b). The flame then slowly reduces until T = 100 s (Fig. 9c,d), where the flame is significantly smaller compared to the previous time instances. These trends are in agreement with the experimental observations. The numerical results indicate that the model is capable of providing a detailed temporal representation of the ignition/extinction, flame spread and the combustion during the pyrolysis process. It also captures the flickering flame motion, which takes into account the air entrainment, buoyancy forces, diffusion and velocity of the burning surface.

**Figure 10:**
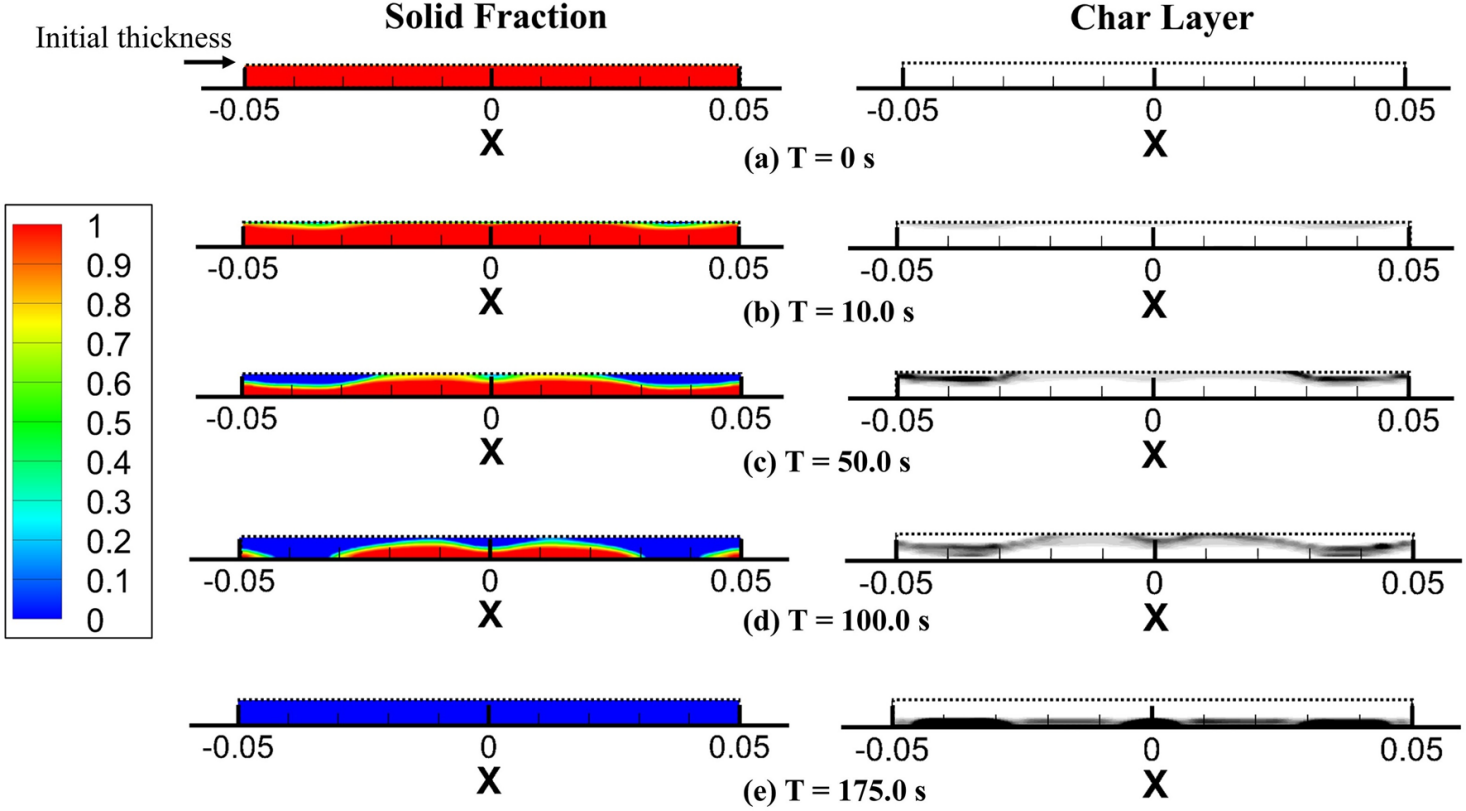
2D contour plot of the solid fraction regression and char layer formation over the burning duration for [CH/PA/Ti$_3\hbox {C}_2$]$_3$ PUF at time instance: (**a**) T = 0 s, (**b**) T = 10.0 s, (**c**) T = 50.0 s, (**d**) T = 100 s, and (**e**) T = 175.0 s.

Figure 10a–e shows the 2D contour plot of the solid fraction regression and char layer formation over the burning duration of [CH/PA/Ti$_3\hbox {C}_2$]$_3$ PUF as pure PUF had no charring residual. The solid mass fraction is represented by a rainbow legend from 0.0 to 1.0, where 1.0 means it is 100% solid and 0.0 means zero solid is left. The char layer is visualised by a greyscale char mass fraction contour from 0.0 to 0.3. The results highlight the capability of the fire model to track the solid material surface interface and the formation of the char layer during cone calorimeter tests. The FR coating reduces the thermal degradation rate of the PUF, thus resulting in a smaller flame and a prolonged burn duration. Furthermore, the enhanced char formation acts as a thermal layer to protect the unburnt virgin material from high temperatures during the pyrolysis process since char has a significantly lower thermal conductivity. A reduction in solid temperature will lead to lower pyrolysis rates and subsequently, a slower HRR. The char layer also decreases the permeability of the solid material surface thus resulting in a shielding effect for the combustible gas volatiles by decreasing the volatile gases to be emitted to the surface. The good agreement between the cone calorimeter test and the model predictions confirms that the CH/PA/Ti$_3\hbox {C}_2$ coating can significantly lower the flammability of PUF by the charring mechanism.

## Conclusion

We have developed and demonstrated a new nanostructured FR coating that can dramatically suppress the flammability of polyurethane foam. Using a LbL method, a two-dimensional transitional metal MXene (Ti$_3\hbox {C}_2$) was hybridised with three different biomass materials (casein, pectin, and PA). Results from a wide range of tests, including cone calorimetry, thermogravimetric, microscale combustion and UL-94 horizontal burning test, reveal that the [CH/PA/Ti$_3\hbox {C}_2$]$_3$ coating is able to reduce pHRR, THR, PSPR, TSR, COPR, and CO$_2$PR by 51.1%, 40.1%, 66.7%, 84.8%, 60.4% and 69.1%, respectively. These unprecedented improvements, which are better than the highest values for PUF reported in the literature, stem from the synergistic effects of the constitutional organic and inorganic compounds engineered with a two-dimensional. In addition, we have presented a high-fidelity CFD to accurately capture the underlying FR mechanisms and predict the outstanding fire and smoke reductions. Using a systematic numerical modelling framework including pyrolysis reaction rate extraction via the genetic algorithm approach and the application of an LES-based fire model, the CFD-based analytical model with an in-house surface regression pyrolysis module accounts for the fundamental thermal decomposition rate and kinetics of FR coated PUFs. Good agreement has been achieved between the computational prediction and the experimental results in terms of HRR, TTI and burning duration. With the incorporation of solid interface tracking and char formation model, the model can replicate the reduction in fire intensity owing to the incorporation of the CH/PA/Ti$_3\hbox {C}_2$ coating. The exceptional performance of the nanostructured CH/PA/Ti$_3\hbox {C}_2$ coating applied on PUF demonstrated significant potential for replacing halogenated FRs with the additional benefit of reduced toxicity and improved biodegradability.

## Methods

### Raw materials

A PUF material with a density of 30 kg/m$^3$ was obtained from Jiangsu Luyuan New Material Co. Ltd., China. Hydrochloric acid (HCl, 36% aq.), Chitosan (CH, Mw. 1500000), casein, pectin, phytic acid (PA, 50% aq.), polyacrylic acid (PAA) and lithium fluoride (LiF) were purchased from Sigma-Aldrich. Titanium aluminum carbide (Ti$_3$AlC$_2$) was purchased from the 11 Technology Co. Ltd., (Changchun, China).

### Preparation of solutions

Six solutions were prepared for the synthesis and coating of MXene hybrids (i.e. CH/casein/MXene, CH/pectin/MXene and CH/PA/MXene). PAA powder was added into deionised (DI) water to form a 0.1 wt% solution. A PA solution was prepared by diluting the PA with DI water to a 2 wt% concentration. CH solution was prepared by dissolving it into DI water with the addition of diluted HCl to maintain a pH value of around 5. Casein was dispersed into DI water by using the phosphate buffer to form 0.5 wt% solution. Pectin solution (0.25 wt%) was prepared by adding the pectin powder into the distilled water. A suspension of Ti$_3\hbox {C}_2$ nanosheets was prepared to a concentration of 1 mg/mL according to our previous work^46^.

### Preparation of layer-by-layer coated PUFs

PUF blocks were cut into samples of 100 mm $\times$ 100 mm $\times$ 25 mm, which were washed thoroughly with DI water and dried in an oven at 60 $^\circ$C for 12 h to a constant weighted before coating. For the LbL deposition, the PUF samples were first put into the polyacrylic acid (PAA) solution for 10 min for the solution to infuse into the foam pores, which was to activate the surface for the subsequent coatings. Then the samples were submerged into the CH, PA, and Ti$_3\hbox {C}_2$ solutions/suspension respectively to synthesise the CH/PA/MXene coating. Each immersion was followed by a washing step, in which the foams were submerged into the DI water to wash off the uncoated materials before the next immersion step. For each monolayer coating, the immersion took around 1 min. This cyclic process was repeated to achieve the desired number of layers; the resultant coating is designated as [CH/PA/Ti$_3\hbox {C}_2$]$_n$, with n denoting the number of layers. As for other combinations, the PA solution was replaced with either casein or pectin solution while all the other steps remained unchanged; the resulting materials are denoted as [CH/Casein/Ti$_3\hbox {C}_2$]$_n$ and [CH/Pectin/Ti$_3\hbox {C}_2$]$_n$. After receiving the desired numbers of coats, the samples were dried in the oven at 70 $^\circ$C for 12 h and then weighed to determine the mass gain before being prepared for characterisations described below.

### Characterisation

Thermogravimetric analysis (TGA) was conducted with a heating rate of 20 K/min under nitrogen gas. The samples were heated from ambient temperature to 900 $^\circ$C. Scanning electron microscopy (SEM) equipped with energy dispersive X-ray spectrometer (EDS) was used to examine the surface morphology of the different types of coated PUFs, identify the element and element distributions on the PUF surfaces, and confirm the quality of coverage by hybrid flame-retardants (FRs). The beam voltage was 5 kV. The samples were coated with carbon prior to the EDS testing. Microscale combustion calorimeter (MCC) tests were performed from 60 $^\circ$C to 750 $^\circ$C at a heating rate of 1 K/s to study the heat release rate (HRR) and total heat release (THR) versus time. The flame retardancy properties were investigated with a cone calorimeter (FTT iCone Classic) at a heat flux rate of 35 kW/m$^2$. The samples were cut into 100 mm $\times$ 100 mm $\times$ 25 mm before testing. UL-94 Horizontal burning test (HF) were conducted according to ASTM D 4986, ISO/DIS 9772.3.

## Supplementary information

Supplementary Information.
