# Supplementary material for: Experimental and numerical perspective on the fire performance of MXene/Chitosan/Phytic acid coated flexible polyurethane foam
Source: Sci Rep. 2021 Feb 25;11:4684. doi: 10.1038/s41598-021-84083-2 (PMC7907131; doi:10.1038/s41598-021-84083-2)
Supplement: Supplementary file 1 — Supplementary Information. [file 41598_2021_84083_MOESM1_ESM.pdf]

Experimental and Numerical Perspective on the Fire Performance of MXene/Chitosan/Phytic Acid Coated Flexible Polyurethane Foam

Bo Lin[1], Anthony Chun Yin Yuen[1,*], Timothy Bo Yuan Chen[1], Bin Yu[3], Wei Yang[1,2], Jin Zhang[1], Yin Yao[1], Shuying Wu[1,4], Chun Hui Wang[1], and Guan Heng Yeoh[1,5,*]

[1]University of New South Wales, School of Mechanical and Manufacturing Engineering, Sydney, NSW 2052, Australia

[2]Hefei University, School of Energy, Materials and Chemical Engineering, Hefei, Anhui 23061, PR China

[3]University of Southern Queensland, Centre for Future Materials, Toowoomba, QLD 4350, Australia

[4]Macquarie University, School of Engineering, Sydney, NSW 2109, Australia

[5]Australian Nuclear Science and Technology Organisation (ANSTO), Kirrawee DC, NSW 2232, Australia

*c.y.yuen@unsw.edu.au; g.yeoh@unsw.edu.au

S1 EDS images of element peaks

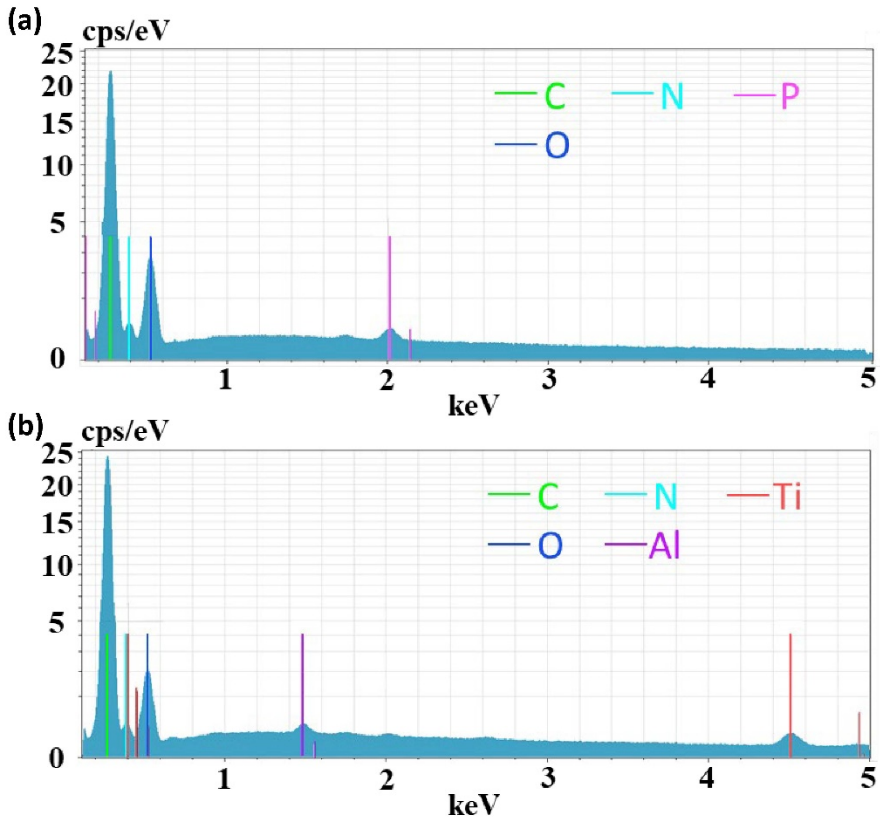

Figure S1. EDS element peak images of (a) $[CH/PA]_1$ coated PUF, and (b) $[CH/PA/Ti_3C_2]_1$ coated PUF.

S2 Summarised TGA and MCC tables

Table S1. Summarised TGA results for the pure and coated PUFs. (20 °C/min, 5-10 mg; errors ± 0.5 wt%, ± 1 °C)

Sample	Weight Gain (wt%)	$T_{5\%}$ (°C)	T_{max1} (°C)	T_{max2} (°C)	Residue at 700 °C (wt%)
Pure Foam	0	248	284	370	0.7
$[CH/Casein/Ti_3C_2]_2$ PUF	9.61	278	310	392	7.3
$[CH/Pectin/Ti_3C_2]_2$ PUF	7.47	268	300	391	5.3
$[CH/PA/Ti_3C_2]_2$ PUF	7.91	264	307	389	12.3
$[CH/PA/Ti_3C_2]_5$ PUF	18.88	267	299	376	28.1

Table S2. Summarised MCC results of pure and all coated PUFs. (error ± 5%)

**Sample**	**pHRR (W/g)**	**pHRR reduction (%)**	**THR (kJ/g)**	**THR reduction (%)**
Pure Foam	413.3	-	25.1	-
$[CH/Casein/Ti_3C_2]_2$ PUF	170.7	59	8.6	66
$[CH/Pectin/Ti_3C_2]_2$ PUF	167.3	59	8.7	65
$[CH/PA/Ti_3C_2]_2$ PUF	112.5	73	7.8	69
$[CH/PA/Ti_3C_2]_5$ PUF	114	72	7.8	69

Table S3. Summarised MCC results of pure and $[CH]_1$, $[CH/Casein]_2$, $[CH/Pectin]_2$ and $[CH/PA]_2$ coated PUFs. (error ± 5%)

**Sample**	**pHRR (W/g)**	**pHRR reduction (%)**	**THR (kJ/g)**	**THR reduction (%)**
Pure Foam	413.3	-	25.1	-
$[CH]_1$ PUF	422.8	-2.3	26.9	-7.2
$[CH/Casein]_2$ PUF	469.0	-13.5	27.4	-9.2
$[CH/Pectin]_2$ PUF	511.3	0.5	27	-7.6
$[CH/PA]_2$ PUF	410.2	0.8	26.5	-5.6

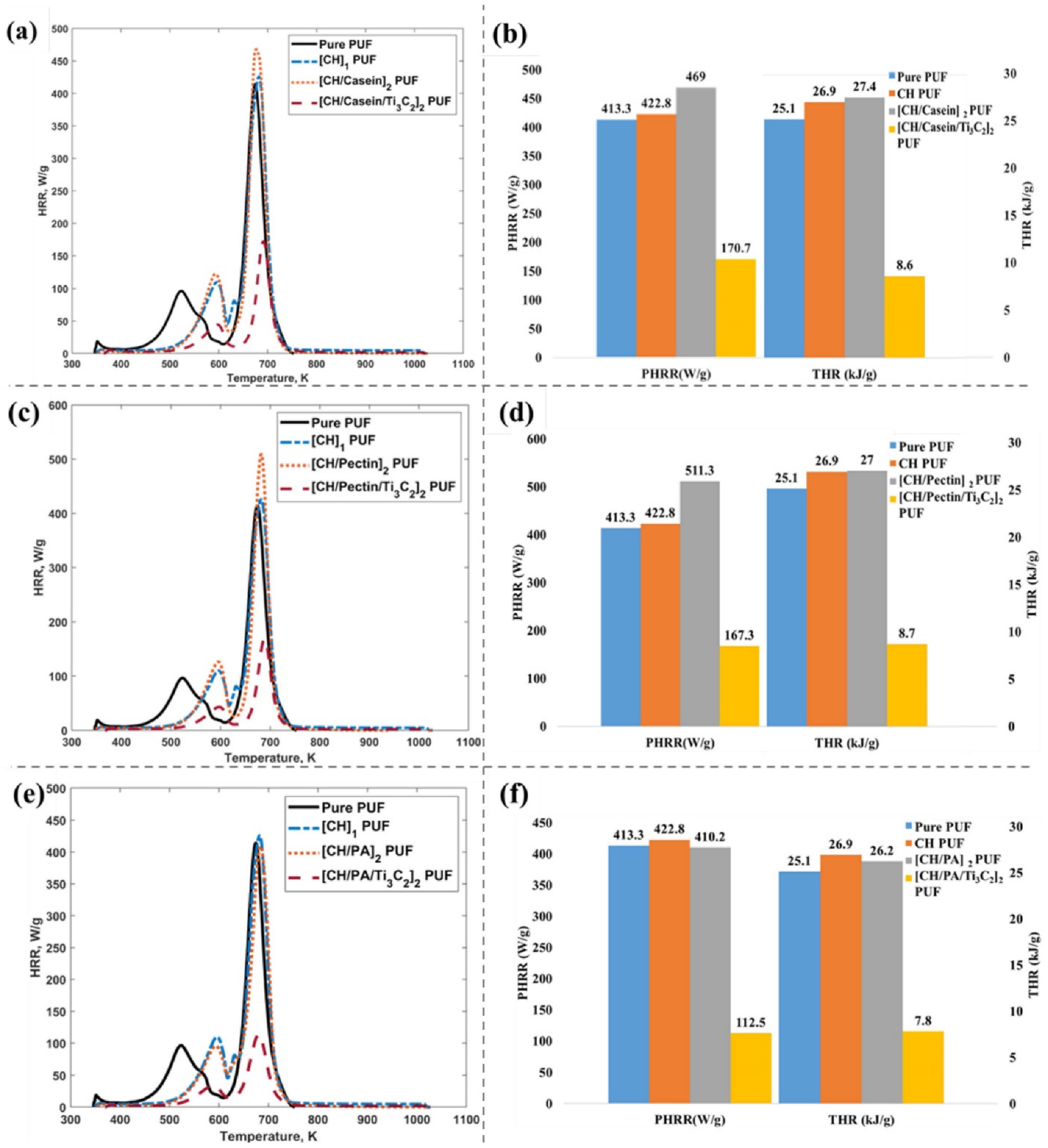

Figure S2. Comparison of biomass material coated PUF MCC results with hybrid coated PUFs MCC results.

Additional MCC tests were conducted to further investigate the synergistic effect between the organic coatings and the inorganic Ti_3C_2 nanosheets. As the layer-by-layer technique requires alternating layers of oppositely charges materials, single layer coatings of the biomass materials including casein, pectin and PA, and Ti_3C_2 nanosheets are not feasible. Consequently, $[CH]_1$,

[CH/Casein]$_2$, [CH/Pectin]$_2$ and [CH/PA]$_2$ coated PUFs were studied via MCC. By comparing the coating with and without Ti_3C_2 (see Fig. S2), it is found that with only organic materials coating (i.e. [CH]$_1$, [CH/Casein]$_2$, [CH/Pectin]$_2$ and [CH/PA]$_2$), there was no substantial improvements to pHRR and THR compared to pure PUF. This is attributed to the combustion of these carbon-rich biomass materials. When the Ti_3C_2 nanosheets were added into the coatings, it results in a dramatic decrease in pHRR and THR compared to its individual components, indicating the synergistic effect of the MXene hybridising coatings. This phenomenon can be attributed to the barrier effect and the promotion of organic char formations by the of Ti_3C_2.

S3 Comprehensive comparison of the current and previous work

Table. S4 summarises the key indicators from the Cone Calorimeter results of the [CH/PA/Ti_3C_2]$_3$ PUF (present work) against [CH/Ti_3C_2]$_5$ PUF (previous work) and pristine PUF. A radar graph comparing the performance of the current and previous work was presented in Fig. S3. The results showed the present CH/PA/Ti_3C_2 coating has a more comprehensive improvement regarding the fire safety parameters compared with the previous work. It should be noted that the effective LbL layers are similar. There are 10 and 9 monolayers for [CH/Ti_3C_2]$_5$ PUF and [CH/PA/Ti_3C_2]$_3$ PUF respectively, whereas the number of MXene layers has been reduced to from 5 to 3, for the [CH/PA/Ti_3C_2]$_3$ PUF. In comparison to CH/Ti_3C_2 coated PUF, the radar graph revealed that the CH/PA/Ti_3C_2 coated PUF achieved higher reductions (i.e. versus pristine PUF) in the peak heat

and gas species production rates including pHRR, PSPR, PCOPR and PCO_2PR. And it should be noted that, there is a significant improvement on the smoke and CO_2 reductions with (-67.6%) and (-67.5%) for $[CH/PA/Ti_3C_2]_3$ PUF, over (-54.5%) and (-57.6%) for $[CH/Ti_3C_2]_5$ PUF, and this can be attributed to the remarkable char formation mechanisms as evidenced in the Char forming performance section.

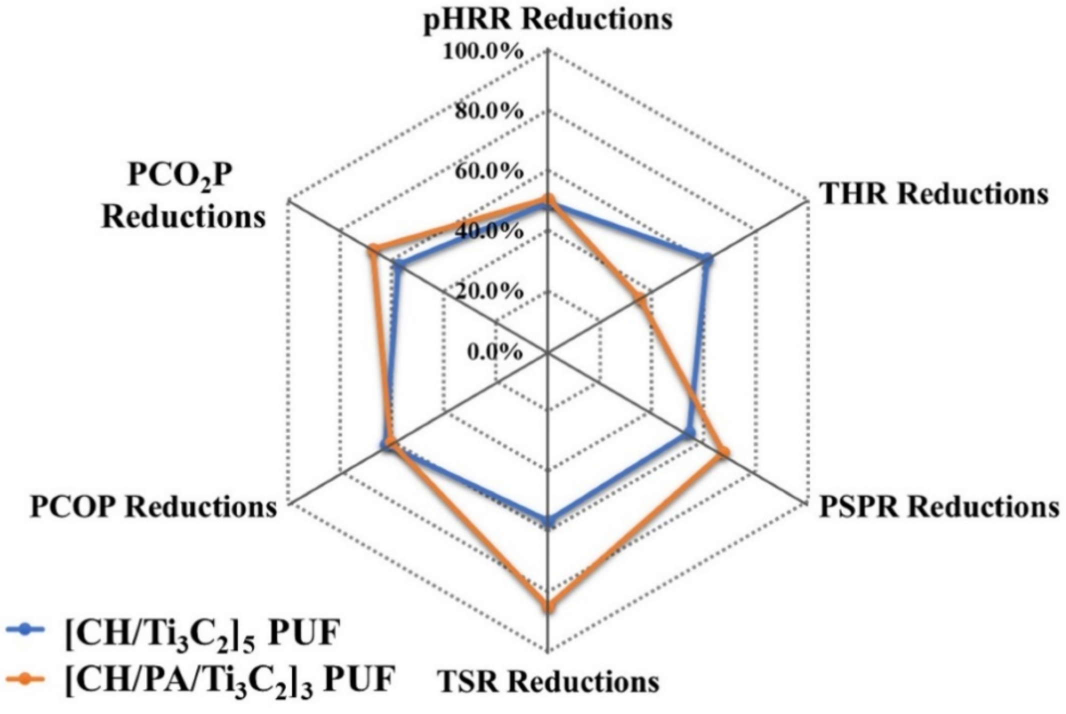

Figure S3. Radar graph of fire safety performance for previous work ($[CH/Ti_3C_2]_5$ PUF) and current work ($[CH/PA/Ti_3C_2]_3$ PUF).

Table S4. Cone calorimeter data of neat PUF and coated PUFs at 35km/m^2. (error ± 5%)

**Sample**	**TTI (s)**	**pHRR (kW/m^2)**	**THR (MJ/m^2)**	**PSPR (m^2/s)**	**TSR (m^2/m^2)**	**PCOPR (g/s)**	**PCO$_2$PR (g/s)**
Pure PUF	5	276	11.9	0.068	173	0.0048	0.191
[CH/Ti_3C_2]$_5$ PUF	5	140	4.6	0.031	75	0.0018	0.081
[CH/PA/Ti_3C_2]$_3$ PUF	6	136	7.7	0.022	26	0.0019	0.062

Fig. S4 summarises the Cone Calorimeter of all-inclusive MXene based LbL assembly coating studies for PUF, where the flame retardancy of our present work is benchmarked against our previous work in terms of the number of MXene monolayers. The six data points depict various fire resistance parameters including pHRR, THR, pSPR, TSR, PCOPR, and PCO$_2$PR. The results for the [CH/PA/Ti_3C_2]$_3$ PUF (present work) were highlighted in red, while the others are results of the [CH/Ti_3C_2]$_5$ PUF based on our previous studies. In comparison to the number of MXene monolayers applied within the polymer matrix, it was identified that our present work significantly outperformed our previous work in terms of pHRR, PSPR, TSR, PCOPR and PCO$_2$PR. This further signified the excellent performances of the MXene biomass nanocomposite. It also confirmed that while MXene was an effective flame-retardant, the optimal amount of monolayer is around 3, while the remaining could be replaced by alternative organic variants to improve the eco-friendliness.

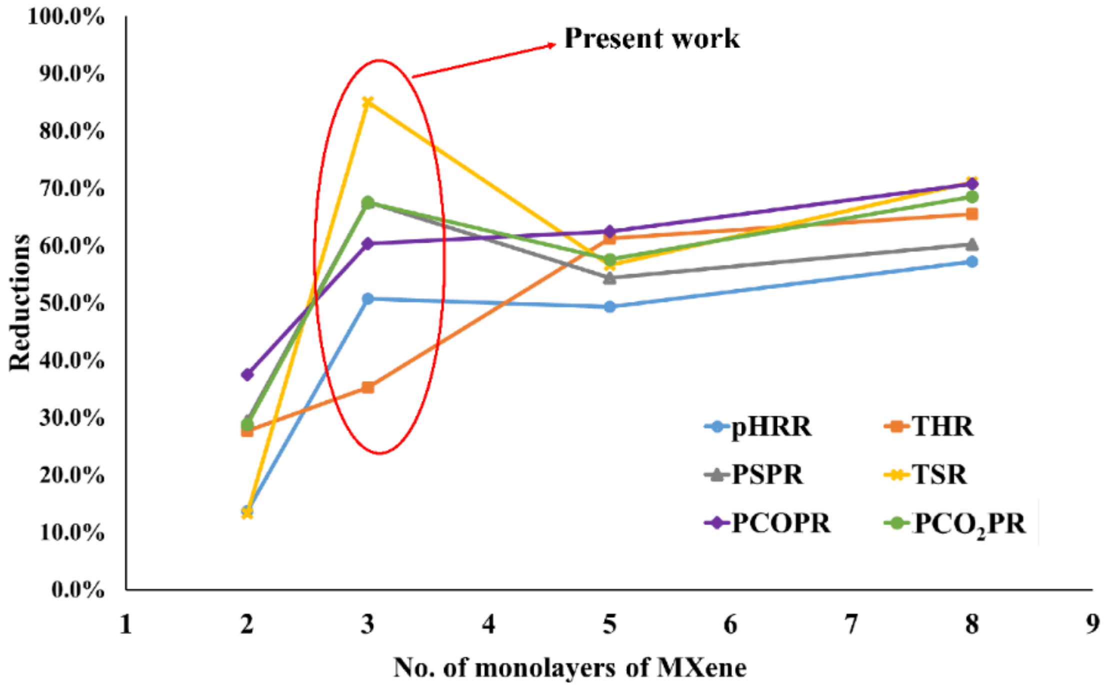

Figure S4. Reductions in pHRR, THR, PSPR, TSR, PCOP, and PCO_2P versus numbers of MXene monolayers (MXene contents).

S4 Numerical results

S4.1 Turbulence and combustion modelling

For turbulence modelling of small-scale eddies (i.e. length scale filter equivalent of the size of the grid), the Smagorinsky subgrid-scale (SGS) model [1] is adopted with a Smagorinsky constant of 0.2 and the turbulent Schmidt and Prandtl numbers are prescribed as 0.5 respectively. These values have been previously validated for various full-scale fire case studies [2-4]. At wall boundaries, the turbulence viscosity term evaluated by the Smagorinsky model was automatically filtered as zero by the wall-damping function.

The strained laminar flamelet combustion model for non-premixed diffusion flames was adopted in this study. When performing LES simulations, the mass fraction of individual species can be determined by a prescribed ''look-up table" in terms of mixture fraction and the variance. In addition, it incorporates detailed chemistry mechanisms (GRI-Mech 3.0 [5]) to simulate the combustion reaction of the emitted gas volatiles. The model allows a more accurate prediction of the oxidation process of the fuel, as well as key partial chemical products including CO, OH, H_2, and C_2H_2 [6]. Furthermore, C_2H_2 could be considered as the main soot precursor in the soot model, which provides a much better representation of the soot nucleation, surface growth, and oxidation processes [7,8].

S4.2 Soot and radiation modelling

In this work, the Moss-Brookes semi-empirical soot model was implemented to account for the soot formation and oxidation where acetylene C_2H_2 is considered as the soot precursor [9]. In this model, the number density and mass fraction of soot particles are solved to reflect the amount of soot concentration within the computational field. The radiative heat transfer is modelled using the filtered radiative transfer equations (FRTE) for non-scattering gray gas solved by the discrete ordinates method (DOM). The Weighted Sum of Gray Gases Model (WSGGM) [10] was used to approximate the filtered gas absorption coefficient for each combustion product as well as the unburned fuel. The overall absorption coefficient was determined as a summation of the coefficients of all the species of the gas mixture and soot. The absorption coefficient for soot particles was determined in terms of the volume fraction and temperature according to Kent and Honnery [11].

S4.3 Numerical characterisation of the thermal degradation rate

The pyrolysis kinetics (e.g. activation energies and pre-exponential factor) were extracted from the derivative thermogravimetric (DTG) data via an iterative MATLAB code [12]. The code consists of firstly, calculating an initial approximation of the pyrolysis kinetics using the Kissinger-Akahira-Sunose (KAS) method [13]. Essentially, the KAS method establishes a linear relationship

between the heating rate β (K/min) and the temperatures $T_{i,p}$ (K) corresponding to reaction peaks in the DTG curve:

$$ln\left(\frac{\beta}{T_p^2}\right) = -\frac{E}{RT_p} + ln\left(\frac{AE}{Rg(x)}\right) \quad (1)$$

where g(x) is the integral conversion function [14], E is the activation energy (J/mol), A is the exponential factor and R is the universal gas constant (8.314 J/mol K).

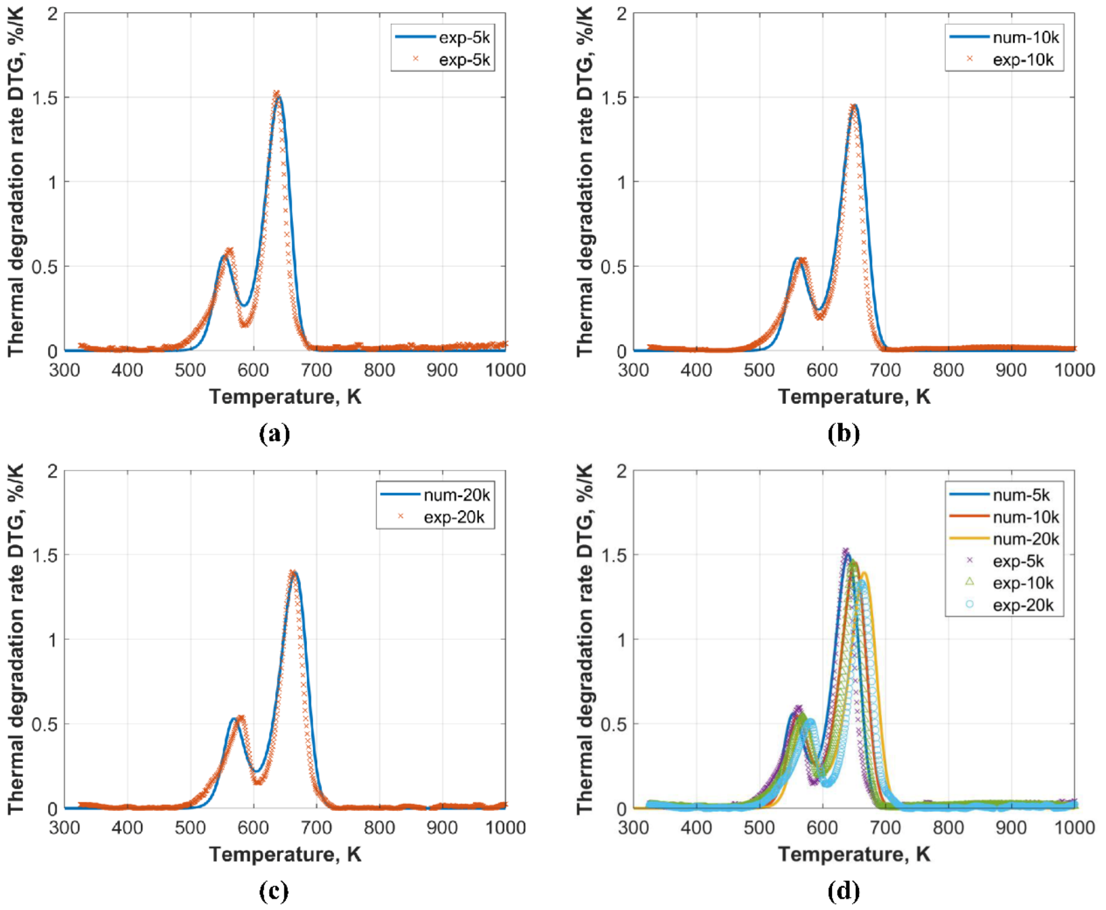

Figure S5. Comparison of numerical results against experimental DTG curves for $[CH/PA/Ti_3C_2]_3$ PUF at (a) 5K/min, (b) 10K/min, (c) 20K/min and (d) Combined.

The slope and intercept of the linear relationship were then used to calculate the activation energy and pre-exponential factor. The initial approximation obtained from the KAS method was then optimised by applying a genetic algorithm (GA) based searching function. The process was repeated after convergence was achieved (i.e. there are no subsequent improvements, or the fitness functions return a value which passes the end criterion of less than 5% error). Fig. S5 shows a comparison between the numerical and experimental DTG curves for $[CH/PA/Ti_3C_2]_3$ PUF.

S4.4 Numerical Setup

Numerical simulations have been performed based on the cone calorimeter experiment using the extracted pyrolysis kinetics. The computational domain of the fire model, consists of the cone geometry and a 60 mm long cylindrical extended region was applied from the cone outlet with a diameter of 80 mm. The domain is illustrated in Fig. S6.

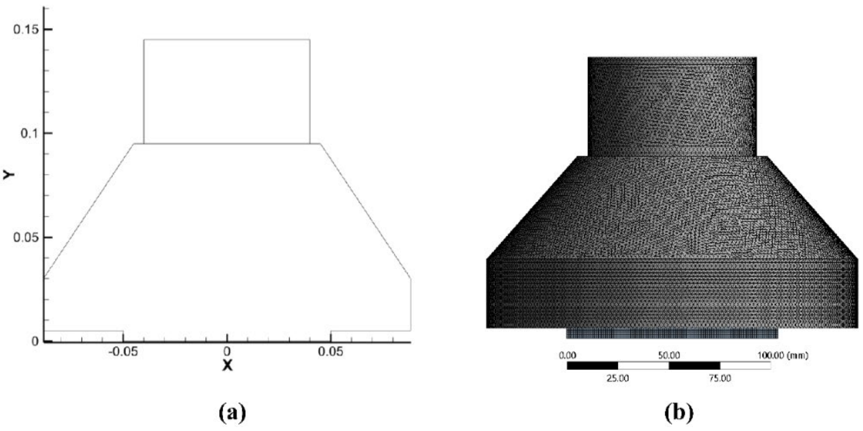

Figure S6. (a) 2D sketch for the cone with dimensions and (b) converted computational domain of the cone geometry in the fire model.

The characteristic length analysis based on DiNenno et al. [15] was applied to determine the appropriate grid system to achieve mesh independence. A fine mesh consisting of an average grid size of 1.5 mm (0.0015 m) was applied in the fire simulations, with a total element of approximately 3210000.

Reference

1 Smagorinsky, J. General circulation experiments with the primitive equations: I. The basic experiment. *Monthly weather review* **91**, 99-164 (1963).

2 Cheung, S., Yeoh, G. H., Cheung, A., Yuen, R. & Lo, S. M. Flickering behavior of turbulent buoyant fires using large-eddy simulation. *Numerical Heat Transfer, Part A: Applications* **52**, 679-712 (2007).

3 Yuen, A. C. Y., Yeoh, G. H., Timchenko, V., Cheung, S. C. P. & Barber, T. J. Importance of detailed chemical kinetics on combustion and soot modelling of ventilated and under-ventilated fires in compartment. *Int. J. Heat Mass Transf.* **96**, 171-188 (2016).

4 Yuen, A. C. Y., Yeoh, G. H., Timchenko, V., Cheung, S. C. P. & Chen, T. Study of three LES subgrid-scale turbulence models for predictions of heat and mass transfer in large-scale compartment fires. *Numerical Heat Transfer; Part A: Applications* **69**, 1223-1241 (2016).

5 Smith, G. P. *et al.* GRI-Mech 3.0. (2000).

6 Yuen, A. C. Y. *et al.* Comparison of detailed soot formation models for sooty and non-sooty flames in an under-ventilated ISO room. *Int. J. Heat Mass Transf.* **115**, 717-729 (2017).

7 Chen, T. B. Y. *et al.* Numerical study of fire spread using the level-set method with large eddy simulation incorporating detailed chemical kinetics gas-phase combustion model. *J. Comput. Sci.* **24**, 8-23 (2018).

8 Chen, T. *et al.* Predicting the fire spread rate of a sloped pine needle board utilising pyrolysis modelling with detailed gas-phase combustion. *International Journal of Heat and Mass Transfer* **125**, 310-322 (2018).

9 Brookes, S. J. & Moss, J. B. Predictions of soot and thermal radiation properties in confined turbulent jet diffusion flames. *Combust. Flame* **116**, 486-503 (1999).

10 Beer, J. M., Foster, P. J. & Siddall, R. G. Calculation methods of radiation heat transfer, HFTS Design Report No. 22, AEA Technology (1971).

11 Kent, J. & Honnery, D. A soot formation rate map for a laminar ethylene diffusion flame. *Combust. Flame* **79**, 287-298 (1990).

12 Yuen, A. C. Y. *et al.* Establishing pyrolysis kinetics for the modelling of the flammability and burning characteristics of solid combustible materials. *Journal of Fire Sciences*, 0734904118800907 (2018).

13 Akahira, T. & Sunose, T. Trans. Joint convention of four electrical institutes. *Res. Rep. Chiba Inst. Technol.* **16**, 22-31 (1971).

14 Idris, S. S. *et al.* Investigation on thermochemical behaviour of low rank Malaysian coal, oil palm biomass and their blends during pyrolysis via thermogravimetric analysis (TGA). *Bioresource Technology* **101**, 4584-4592 (2010).

15 DiNenno, P. J., Drysdale, D., Beyler, C. L. & Walton, D. W. *SFPE handbook of fire protection engineering*. 3rd edition edn, (National Fire Protection Association, 2002).
